# Wheat Allergy in Children: A Comprehensive Update

**DOI:** 10.3390/medicina55070400

**Published:** 2019-07-23

**Authors:** Giampaolo Ricci, Laura Andreozzi, Francesca Cipriani, Arianna Giannetti, Marcella Gallucci, Carlo Caffarelli

**Affiliations:** 1 Pediatric Unit, Department of Medical and Surgical Sciences, University of Bologna, 40139 Bologna, Italy; 2 Clinica Pediatrica, Department of Medicine and Surgery, University of Parma, 43126 Parma, Italy

**Keywords:** anaphylaxis, asthma, food allergy, recombinant allergen, *Triticum aestivum*, wheat

## Abstract

Gluten-related disorders are very common in pediatric patients. Wheat allergy is triggered by an immunoglobulin E (IgE)-dependent mechanism; its prevalence varies according to the age and region, and in Europe has been estimated to be lower than 1%. Many studies investigated the potential role of several external factors that can influence the risk to developing wheat allergy, but results are still inconclusive. It can be responsible for several clinical manifestations depending on the route of allergen exposure: food-dependent exercise-induced anaphylaxis (FDEIA), occupational rhinitis or asthma (also known as baker’s asthma), and contact urticaria. The prognosis of IgE-mediated wheat allergy in children is generally favorable, with the majority of children becoming tolerant by school age. Patients who experienced an anaphylactic reaction prior to 3 years of age and patients with higher level of wheat- or ω-5 gliadin-specific IgE antibodies seem to be at higher risk of persistent wheat allergy. The current management of patients is dietary avoidance. Nowadays, oral immunotherapy has been proposed for wheat allergy with promising results, even if further studies are necessary to establish the best protocol in order to promote tolerance in wheat-allergic children.

## 1. Introduction

Food allergies are widespread all over the world, with a prevalence up to 10% in Western countries among infants and an increasing prevalence in developing countries [1]. Food allergies are more common in children than adults. Few foods account for over 80% of the reaction in food allergies: milk, egg, soy, wheat, peanut, and tree nuts [1,2].

Wheat (*Triticum aestivum*) is the most widely consumed food grain in the world thanks to its ability to grow in different climatic areas. It can be responsible of a wide range of disorders, depending on the route of allergen exposure and the underlying immunologic mechanisms.

**Figure 1:**
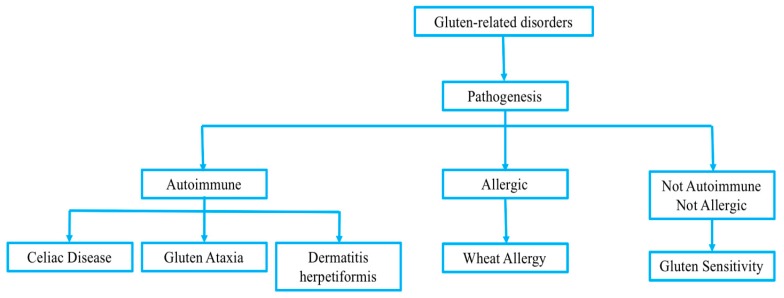
Gluten-related disorders classification.

Gluten-related disorders are very common in pediatric patients. Recently, celiac disease (CD), wheat allergy (WA), and gluten sensitivity have been included in the spectrum of gluten-related disorders, even if their pathogenesis is deeply different (Figure 1) [3].

CD is an autoimmune disorder that in most countries is treated according to gastroenterological protocols, WA is usually triggered by an IgE-dependent mechanism, while gluten sensitivity is considered separately because it is neither an autoimmune, nor an allergic disease. 

Wheat is one the most common food allergens in children. It can be responsible of several clinical manifestations: food-dependent exercise-induced anaphylaxis (FDEIA), occupational asthma (or Baker’s asthma) and rhinitis, or contact urticaria [4].

## 2. Epidemiology

The prevalence of WA varies according to the age and region [5]. In most countries, cow’s milk allergy and egg allergy represent the two most common single allergies, but wheat comes as third at least in Germany, Japan, Finland [6], and in preschool children in US [7]. In Europe, a WA prevalence <1% has been reported in different studies. A systematic review conducted by Zuidmeer et al. in 2008 showed that prevalence of WA, confirmed by oral food challenge (OFC) that is the diagnostic standard, varied between 0.2% and 0.5% in patients younger than 14 years [8]. In UK, a prevalence of 0.48% has been reported in children, according to positive OFC [9]. In a cohort study, Osterballe et al. [10] reported that none of the children selected had a history of symptoms after wheat ingestion. Ostbloom et al. reported that prevalence of WA was 4% in 4 year old patients [11]. 

In a systematic review conducted by Nwaru et al. [12], a prevalence of positive wheat challenges was 0.1%. Considering both the history of wheat allergy and positive OFC results, the prevalence increases to 0.3%. Many studies confirmed that the self-reported prevalence of WA is higher than WA ascertained by OFC. Nwaru et al. [12] found a lifetime and point self-reported prevalence of 3.6% and 1.5%, respectively. Similarly, Zuidmeer et al. [8] confirmed that in children younger than 15 years of age, the perception of allergic reactions to wheat (0.2–1.3%) was slightly higher than the positive wheat challenge. However, in Italian school children, self-reported WA had a prevalence of 9% [13]. It is recognized that self-reporting usually overestimates food allergy prevalence by a factor of three to four [14]. This difference between self-reported and challenge-verified prevalence of WA can be due to parents mistaking other adverse reactions to food (i.e., gluten sensitivity) for food allergy [12]. The prevalence of positive skin tests to wheat ranged from 0.2% to 1%, while prevalence of positive serum IgE antibodies to wheat ranged from 0.4% to 3.6% in patients younger than 14 years [8]. Among American children, data from skin prick test (SPT) suggest a WA prevalence higher than 3%, even if it is more likely estimated to be 0.2% to 1% [15,16,17]. In Australian children, Hill et al. found that prevalence hypersensitivity to wheat was 0.15% [18]. In a general population of Japanese children aged 0–6 years, the prevalence of positive wheat SPT/ω-5 gliadin serum IgE antibodies was 0.37% in healthy population [19]. In Asia–Pacific children with allergic symptoms, the prevalence of positive wheat SPT/wheat serum IgE antibodies varied from 10.4% to 26.1% [20]. Prevalence of positive SPTs to foods is higher in children with atopic dermatitis [21]. In Italy, a rate of 0.7% has been reported among children aged 9 and 13 years, according to positive Atopy Patch Test (APT) results [22].

### 2.1. Wheat Allergens

Wheat belongs to the grass family Poaceae and contains many allergenic proteins, divided in four classes on the basis of extraction in a series of solvents. This classification was formalized by the American chemist T. B. Osborne in the 19th century [23]. He described four wheat protein fractions: albumins (extracted in water), globulins (extracted in dilute saline), gliadins (extracted in alcohol/water mixtures), and glutenins (extracted in dilute acid). The latter two classes account for 85% of wheat proteins and are known as gluten or prolamine, because of their high proline content [24]. The gliadins are classified into three groups on the basis of their electrophoretic mobility at low pH: these are α/β-gliadins (fast), γ-gliadins (intermediate), and ω-gliadins (slow).

The glutenins are classified into high-molecular-weight (HMW) and low-molecular-weight (LMW) groups after separation by electrophoresis [25].

Many wheat-allergic individuals have been shown to be sensitized to α, β, γ, and/or ω-globulins and to high- and low-molecular-weight glutenins [26,27].

Wheat contains albumin/globulin that can be responsible for WA. They include: β-amylase, inhibitors of hydrolytic enzymes (notably α-amylase and proteinases) and surface-active proteins such as nonspecific lipid transfer proteins (LTPs) and puroindolines. Several allergens (primarily α-amylase inhibitor and LTPs) cross-react with grass pollen allergens, as wheat is a grass from the Poaceae family [28]. Table 1 shows the main wheat allergens responsible for symptoms of WA.

α-Amylase inhibitors and tripsin inhibitors are heat-resistant allergens and are commonly involved in Baker’s asthma, anaphylaxis, and also in wheat-dependent exercise-induced anaphylaxis (WDEIA). Tri a 14 belongs to the nsLTP group and it has been shown to be an important allergen for IgE-mediated food allergies (especially in Italian children), WDEIA, and Baker’s asthma. Tri a 19 is a water-insoluble ω-5-gliadin and it has been identified as a major allergen in subjects with WDEIA. Tri a 37 is a plant defense protein, highly stable and resistant to heat and digestion; patients who have IgE antibodies against Tri a 37 have a four-fold increased risk of severe allergic symptoms upon wheat ingestion [15].

### 2.2. Clinical Manifestations 

WA clinical manifestations can be different depending on the route of allergen exposure.

Wheat ingestion is usually responsible for typical IgE-mediated reactions, with the development of symptoms within 2 h after the ingestion. Urticaria, angioedema, bronchial obstruction, nausea, abdominal pain, or systemic anaphylaxis can occur [29] after the ingestion of wheat proteins [25]. FDEIA is also a manifestation of WA. It is a severe allergic reaction induced by the ingestion of a causative food and subsequent physical exercise. The pathophysiological mechanisms underlying this disease have not been fully demonstrated, but the combination of food and exercise is necessary to elicit the reaction. Clinical manifestations can vary from urticaria/angioedema to severe allergic reactions including severe anaphylaxis; symptoms occur 1–6 h after wheat ingestion followed by physical exercise. It is known that WDEIA is associated with positive IgE antibodies to ω-5-gliadins (Tri a 19) in about 80–90% of patients, even if patients are usually sensitized to several wheat allergens [25].

Food protein-induced enterocolitis syndrome is a non-IgE-mediated manifestation. Infants develop vomiting, pallor, and lethargia 1–4 h following ingestion of wheat in the absence of IgE-mediated skin or respiratory symptoms [30]. Wheat is also a common eliciting factor of eosinophilic esophagitis [31]. Irritable bowel syndrome and constipation are not associated with allergy [32]. Baker’s asthma and rhinitis result from the inhalation of wheat and cereal flours. Baker’s asthma is recognized as one of the most common types of occupational asthma [33] and seems to be associated to allergic sensitization to α-amylase inhibitor proteins [25] and wheat LTP [34].

Contact urticaria is due to hydrolyzed wheat proteins contained in cosmetics and develops after their application to the skin.

### 2.3. IgE Tests

The result of SPT/IgE to wheat may indicate the risk of having WA in children with possible clinical hypersensitivity reactions to wheat. Sensitivity of SPT to fresh wheat or wheat extracts varies from 0.69 to 0.89, specificity from 0.64 to 0.77, positive predictive value from 0.56 to 0.86, and negative predictive value from 0.76 to 0.97 [5,35,36,37]. Sensitivity of serum IgE antibodies to wheat extracts varies from 0.62 to 0.97, specificity from 0.58 to 1.00, positive predictive value from 0.41 to 1.00, and negative predictive value from 0.60 to 0.97 [5,24,36,38,39,40]. In the last few years, component-resolved diagnosis has been applied also in the diagnosis of IgE-mediated WA and WDEIA, with the effort to find single components predicting clinical reactivity. Commercial tests are available to measure IgE sensitization to Tri a 14, Tri a 19, and gliadins. Although an early study showed up to 100% specificity for clinical response with sensitization to ω-5-gliadin (Tri a 19), later, larger studies recruiting more heterogeneous patient groups showed that its sensitivity varies from 0.44 to 0.78, specificity from 0.79 to 0.97, positive predictive value from 0.81 to 0.93, and negative predictive value from 0.62 to 0.69 [24,40,41,42]. Measuring sensitization to Tri a 14 (nsLTP) may help in differentiating wheat sensitization from pollen allergy in patients with high levels of grass pollens-specific IgE, but with a low sensitivity. Therefore, component resolved diagnosis (CRD) does not significantly improve the accuracy of measuring the risk for developing a reaction to wheat, as ascertained by SPT/IgE to extracts [5,24,43], so that the precise diagnosis still relies on specific clinical standardized challenges done under medical supervision.

### 2.4. Risk Factors for Wheat Allergy

The risk of developing food allergy is firstly influenced by the genetic background of the patient. For this reason, food allergies, similarly to atopic dermatitis and asthma, are more likely to occur in infants with a family history of atopic disease [44]. In children with wheat allergy, atopic disorders often coexist, including atopic dermatitis (53–87%), asthma (48–75%), allergic rhinitis (34–62%). About 90% of infants have been reported to be allergic to other foods. Cow’s milk and/or egg are more frequently associated with WA, less frequently fish, soya, and nuts [26,45,46,47]. Sensitization to grasses is associated with an increased risk for occurrence of sensitization to wheat over time [48]. In children with positive IgE to Phl p12 (profilin) and to MUXF3 CCD (Cross-reactive Carbohydrate Determinant), the grass–wheat cross-reactivity seems to be more common [43].

Furthermore, a wide number of environmental factors can affect this risk of developing WA [49]. Many studies focused on factors, but results are inconclusive.

#### 2.4.1. Timing of Initial Exposure to Cereal Grains 

The timing of introduction of cereal grains during weaning and risk of developing WA has been a long-debated topic. Historically, early exposure to solid foods in infancy has been related to the development of allergy, even if there were no well-designed studies to demonstrate the risk of such advice [50,51]. In 2006, Poole et al. [17] showed that, conversely, delaying exposure to wheat until after 6 months was associated with a higher risk (>4 times) of developing WA.

This finding influenced the subsequent statement by the European Society for Paediatric Gastroenterology Hepatology and Nutrition (ESPGHAN) in 2008, which recommended to avoid both early (less than 4 months) and late (7 or more months) introduction of gluten because this might reduce the risk of WA [52].

In 2010, Nwaru et al. [53] conducted a large study including 994 children with HLA-conferred susceptibility to type 1 diabetes mellitus. The authors demonstrated that sensitization to wheat allergen was related to late (>6 months) introduction of wheat in children’s diet.

Genetic factors can influence the relationship between primary exposure to allergen and risk of food sensitization. In a US cohort, complementary food introduced <4 months was associated with a reduced risk of allergen sensitization by the age of 2 to 3 years, but only for children with a parental history of asthma or allergy [54]. The authors suggested that parental history of asthma or allergy may be a marker for the infant’s reduced ability to develop tolerance on exposure to large quantities of food proteins at a time when the gut and immune system are immature [54].

A recent systematic review including seven studies showed that earlier introduction of wheat or gluten most likely lowers the risk of wheat sensitization early in life, but it does not affect the risk of WA [49].

According to the Australasian Society of Clinical Immunology and Allergy (ASCIA) guidelines, wheat should be introduced within the 12th month of age in order to reduce the risk of food allergy [55].

The European Academy of Allergy and Clinical Immunology (EAACI) recommend to introduce complementary foods after the age of 4 months, according to normal standard weaning practices and nutrition recommendations, for all children irrespective of atopic heredity [56]. We suggest that in infants at high risk of allergies, weaning should start at about six months of age, according to cultural traditions as advised by the World Health Organization (WHO). All foods can be progressively introduced into the diet in the first year of life. The available evidence does not support recommendations that either avoidance or exposure to allergenic foods during infancy may prevent food allergy [57].

#### 2.4.2. Breastfeeding

Breastfeeding has also been studied as a risk or protective factor in developing WA. Since the 1930s, many studies have examined the benefits of breastfeeding on the development of atopic diseases [44]. Many studies concluded that breastfeeding seems to protect from the development of atopic disease, and this protective effect appears even stronger in children with a familial history of atopy [44,58].

On the contrary, according to a meta-analysis of meta-analyses published by Victora et al., there is no evidence of an association between breastfeeding and food allergies [59].

Maternal dietary exposure during pregnancy and lactation seems not to contribute significantly to the development of food allergy in the infant [44]. Although there is no clear explanation about the association between the maternal dietary exposure and infant food allergies, because of the finding of many food antigens in human milk, some authors still recommend a maternal free diet [60].

Breastfeeding seems to have no effect on the risk of developing WA [9,17].

According to some studies, breastfeeding during the introduction of complementary foods is important for promoting tolerance [61]. On the contrary, according to Poole et al., breastfeeding when first exposed to cereal grains did not influence the risk of WA; however, increased breastfeeding duration was associated to a higher risk of WA [17]. Similarly, in a case–control study, a longer duration (>6 months) of breastfeeding was associated with a higher risk of WA [62].

A study conducted by Besednjak-Kocijancic [63] found that exclusive breastfeeding is a protective factor for WA at 1 year.

To date, according to a recent metanalysis, the correlation between breastfeeding and WA is not clearly explained [49].

### 2.5. Risk Factors for Persistent Wheat Allergy

Despite the high prevalence of WA in childhood, relatively little is known about its natural history. The prognosis of IgE-mediated WA in children is generally favorable, with 45–69% of children becoming tolerant by 6 years of age [45,46,47,64]. Keet et al. found in a large population of wheat allergic patients (mean age 19 months, range 11–42 months) that the median age at resolution was approximately 6 1⁄2 years. By 4 years of age, 29% had become tolerant, and by the age of 10 years, 62% had become tolerant. Thirty-five percent remained allergic into their teenage years [45].

A recent Japanese retrospective cohort study evaluated the factors associated with persistent WA. Eighty-three children who had a history of immediate-type allergic reaction to wheat were enrolled and were followed until 6 years of age. The rates of tolerance acquisition, evaluated through OFC method, at 3, 5, and 6 years of age were 20.5%, 54.2%, and 66.3%, respectively. Children who were at higher risk of persistent WA were: patients who experienced an anaphylactic reaction prior to 3 years of age in response to all foods and to wheat and patients with a high level of wheat- or ω-5 gliadin-specific IgE antibodies [64]. Tolerance to wheat develops in 76–96% of patients at 16–18 years of age [26,46]. On the other hand, cross-reactivity between grass pollen and wheat that is probably linked to some panallergenes, Phl p 12, profilin, CCD [43], causes an increased risk of sensitization to wheat, but not of WA over time in children [48].

## 3. New Perspectives

The current management for WA is dietary avoidance. Nowadays, oral immunotherapy (OIT) has been proposed for WA, with promising results.

To date, only few studies assessed the efficacy and the safety of OIT for WA, as summarized in Table 2 [4,65,66,67,68].

A recent randomized controlled trial assessed the efficacy and safety of vital wheat gluten (VWG) OIT in 46 patients with wheat allergy aged 4.2–22.3 years [4]. Authors concluded that low- and high-dose VWG OIT induced desensitization in about 50% of the subjects after 1 year of treatment. Two years of low-dose VWG OIT resulted in 30% desensitization, and 13% had sustained unresponsiveness.

According to these preliminary results, OIT appears to be an effective new therapeutic approach in children diagnosed with WA, even if, in terms of safety, it is a risk-taking approach. Among 7822 low-dose VWG OIT doses in the first year, 15.4% were associated with adverse reactions: 0.04% were severe, and 0.08% subjects received epinephrine [4]. Therefore, the choice to start OIT for WA must always be guided by a careful assessment of the risk/benefit ratio. Further studies are necessary to establish the best wheat OIT protocol in order to promote tolerance in wheat-allergic children.

## 4. Conclusions

WA is a quite common allergy among pediatric patients and can be responsible for several clinical manifestations depending on the route of allergen exposure. The prognosis of IgE-mediated wheat allergy in children is generally favorable. CRD may be useful in predicting persistent WA. The current management of patients is dietary avoidance, but nowadays oral immunotherapy has been proposed for wheat allergy, with promising results, even if further studies are necessary to establish the best protocol in order to promote tolerance in wheat-allergic children.

## Figures and Tables

**Table 1:** Main wheat allergens.

Allergen	Common Name	Sources	Tissues	Routes of Exposure
Tri a 12	Profilin	Grasses, Plants, Poaceae, Triticum aestivum, Wheat	Pollen, Seed	Ingestion, Inhalation
Tri a 14	Lipid Transfer Proteins	Grasses, Plants, Poaceae, Triticum aestivum, Wheat	Seed	Ingestion, Inhalation
Tri a 15	alpha-Amylase Inhibitors	Grasses, Plants, Poaceae, Triticum aestivum, Wheat	Seed	Inhalation
Tri a 18	Agglutinins	Grasses, Plants, Poaceae, Triticum aestivum, Wheat	Seed	Ingestion
Tri a 19	ω-5 gliadins	Grasses, Plants, Poaceae, Triticum aestivum, Wheat	Seed	Ingestion
Tri a 20	γ-gliadins	Grasses, Plants, Poaceae, Triticum aestivum, Wheat	Seed	Ingestion
Tri a 21	α-β-gliadins	Grasses, Plants, Poaceae, Triticum aestivum, Wheat	Seed	Ingestion
Tri a 25	Thioredoxin	Grasses, Plants, Poaceae, Triticum aestivum, Wheat	Seed	Ingestion
Tri a 26	Glutenins	Grasses, Plants, Poaceae, Triticum aestivum, Wheat	Seed	Ingestion
Tri a 27	Thiol Reductase	Grasses, Plants, Poaceae, Triticum aestivum, Wheat	Seed	Inhalation
Tri a 28	α -amylase Inhibitor	Grasses, Plants, Poaceae, Triticum aestivum, Wheat	Seed	Ingestion, Inhalation
Tri a 29	α -amylase Inhibitor	Grasses, Plants, Poaceae, Triticum aestivum, Wheat	Seed	Inhalation
Tri a 30	α -amylase Inhibitor	Grasses, Plants, Poaceae, Triticum aestivum, Wheat	Seed	Ingestion, Inhalation
Tri a 31	Triosephosphate Isomerases	Grasses, Plants, Poaceae, Triticum aestivum, Wheat	Seed	Inhalation
Tri a 32	Peroxiredoxines	Grasses, Plants, Poaceae, Triticum aestivum, Wheat	Seed	Inhalation
Tri a 33	Trypsin Inhibitors	Grasses, Plants, Poaceae, Triticum aestivum, Wheat	Seed	Ingestion, Inhalation
Tri a 34	Glyceraldehyde-3-phosphate dehydrogenases	Grasses, Plants, Poaceae, Triticum aestivum, Wheat	Seed	Inhalation
Tri a 35	Dehydrins	Grasses, Plants, Poaceae, Triticum aestivum, Wheat	Seed	Inhalation
Tri a 36	Glutenins	Grasses, Plants, Poaceae, Triticum aestivum, Wheat	Seed	Ingestion
Tri a 37	Thionins	Grasses, Plants, Poaceae, Triticum aestivum, Wheat	Seed	Ingestion

**Table 2:** Clinical studies on efficacy and safety of oral immunotherapy for wheat allergy.

References	Patients, n	Age, Mean (Range)	Duration Up-Dosing Phase	Duration Manteinance Phase	Target Dose	Efficacy, % Desensitized Pts	Adverse Reaction (% Doses)
del Rio et al. 2014 [65]	6	5.5 yrs (5–11)	3–24 days	6 months	12.52 g of wheat protein	83	6.25 (up-dosing phase)
Sato et al. 2015 [66]	18 anaphylactic WA pts	9 yrs (5.9–13.6)	5 days	>3 months	5.2 g of wheat protein	83.3	26.4% (up-dosing phase)
Khayatzadeh et al. 2016 [67]	8 anaphylactic WA pts	7 yrs (5.5–19)	4.6 days (RUSH method)	3 months	5.2 g of wheat protein	92.3	29.6 (up-dosing phase)
	5 non-anaphylactic pts		72.4 days (outpatient method)				2.5 (up-dosing phase)
Rekabi et al. 2017 [68]	12 anaphylactic WA pts	2.25 yrs (2–10)	6.5 months	18 months	70 g spaghetties	100	0.06
Nowak-Wegrzyn et al. 2019 [4]	46 WA pts (23 active group)	8.7 yrs (4.2–22.3)	44 weeks	8 weeks–14 months	4443 mg of wheat protein	52.2 at year 1 30.4 at year 2	15.4% (year 1)

## Abbreviations

CD	Celiac Disease
WA	Wheat Allergy
FDEIA	Food-Dependent Exercise-Induced Anaphylaxis
OFC	Oral Food Challenge
APT	Atopy Patch Test
SPT	Skin Prick Tests
HMW	High Molecular Weight
LMW	Low Molecular Weight
LTPs	Lipid Transfer Proteins
WDEIA	Wheat-Dependent Exercise-Induced Anaphylaxis
ASCIA	Australasian Society of Clinical Immunology and Allergy
VWG	Vital Wheat Gluten
OIT	Oral Immunotherapy
